# Application of Artificial Intelligence in Clinical Dentistry, a Comprehensive Review of Literature

**DOI:** 10.30476/dentjods.2023.96835.1969

**Published:** 2023-12-01

**Authors:** Kimia Ghods, Arash Azizi, Aryan Jafari, Kian Ghods

**Affiliations:** 1 Student of Dentistry, Membership of Dental Material Research Center, Tehran Medical Sciences, Islamic Azad University, Tehran, Iran; 2 Dept. Oral Medicine, Faculty of Dentistry, Tehran Medical Sciences, Islamic Azad University, Tehran, Iran; 3 Student of Dentistry, Membership of Dental Material Research Center, Tehran; 4 Dept. of Mathematics and Industrial Engineering, Polytechnique Montreal, Montreal, Canada

**Keywords:** Artificial Intelligence, Dentistry, Machine learning, Deep learning, Diagnostic System

## Abstract

**Statement of the Problem:**

In recent years, the use of artificial intelligence (AI) has become increasingly popular in dentistry because it facilitates the process of diagnosis and clinical decision-making. However, AI holds multiple prominent drawbacks, which restrict its wide application today. It is necessary for dentists to be aware of AI's pros and cons before its implementation.

**Purpose:**

Therefore, the present study was conducted to comprehensively review various applications of AI in all dental branches along with its advantages and disadvantages.

**Materials and Method:**

For this review article, a complete query was carried out on PubMed and Google Scholar databases and the studies published during 2010-2022 were collected using the keywords "Artificial Intelligence”, "Dentistry," "Machine learning," "Deep learning," and "Diagnostic System." Ultimately, 116 relevant articles focused on artificial intelligence in dentistry were selected and evaluated.

**Results:**

In new research AI applications in detecting dental abnormalities and oral malignancies based on radiographic view and histopathological features, designing dental implants and crowns, determining tooth preparation finishing line, analyzing growth patterns, estimating biological age, predicting the viability of dental pulp stem cells, analyzing the gene expression of periapical lesions, forensic dentistry, and predicting the success rate of treatments, have been mentioned. Despite AI's benefits in clinical dentistry, three controversial challenges including ease of use, financial return on investment, and evidence of performance exist and need to be managed.

**Conclusion:**

As evidenced by the obtained results, the most crucial progression of AI is in oral malignancies' diagnostic systems. However, AI's newest advancements in various branches of dentistry require further scientific work before being applied to clinical practice. Moreover, the immense use of AI in clinical dentistry is only achievable when its challenges are appropriately managed.

## Introduction

John McCarthy introduced the term artificial intelligence (AI) in 1956, and it is defined as " a field of science and engineering involved with the machine understanding of what's usually known as intelligent behavior, and with the creation of artifacts that manifest such behavior" [ 1 ] In other words, AI is a technology that enables machines to perform tasks usually performed by humans [ 2 ].

**Figure 1:**
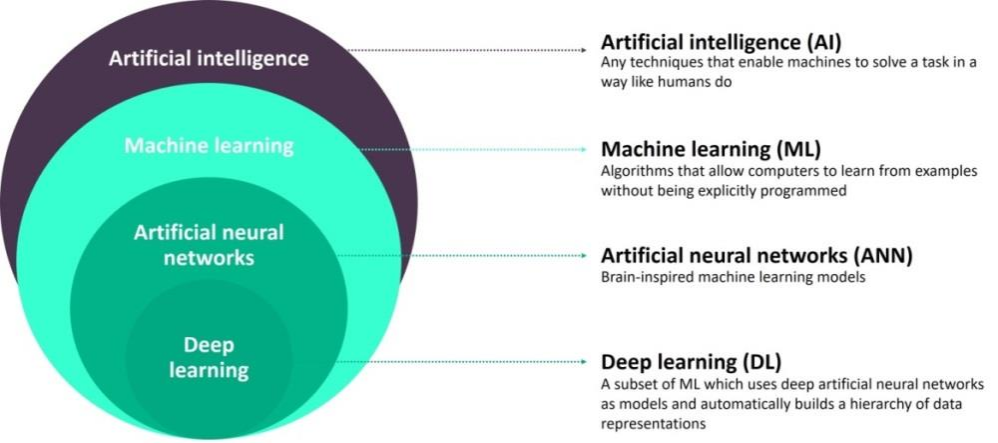
A schematic view of AI, ML, ANN, and DL

Numerous forms of AI in dentistry have been introduced in the last two decades. The application of AI in dentistry initiated with one of its most prevalent types, which was machine learning (ML). In ML, the aim was to design a machine/ system using algorithms in a way that it could learn and operate without explicitly planning and dictating each action. [ 3 - 4 ]. Along with rapid advancements of AI in dentistry, another type of AI named artificial neural networks (ANN) was introduced. This algorithm sought to develop information processing inspired by the human brain's neural network. In other words, the neural network helped train computers to respond appropriately to events, instead of dictating what needs to be done. Each neuron in this network is a processing element and solves different problems along with other processing elements [ 5 - 6 ]. Probably the newest development of AI systems in dentistry is deep learning (DL). This system uses several different layers of neural networks. Each of these layers analyzes parts of the input information. Therefore, this mechanism predicts outcomes based on unlabeled and unstructured data [ 6 - 9 ]. Figure 1 shows a schematic view of AI, ML, ANN, and DL. 

Numerous studies have reported multiple advantages achieved using AI technology in dentistry. The most eminent advantage of AI is probably its capability to integrate and cross-link data collected from imagery techniques with non-imagery data, including clinical records and general and dental history data, to result in better diagnosis. Nevertheless, the immense application of AI is still a matter of discussion, as its possible drawbacks have been mentioned in new research [ 2 ]. Further, in this review article, we will discuss applications of AI in different branches of dentistry along with its challenges.

## Materials and Method

**Figure 2:**
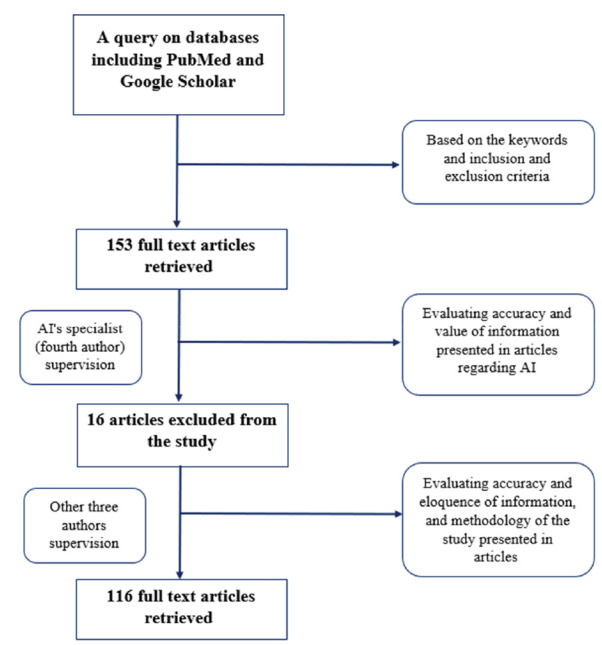
A schematic chart of article's methodology

A query on some databases, such as PubMed and Google Scholar, was carried out using “Artificial Intelligence”, “Dentistry”, “Machine learning”, “Deep learning”, and “Diagnostic Systems “as keywords. In view of the fact that the application of AI in dentistry has started in the last 2 decades, articles published between the years 2010 and 2022 were selected. Moreover, based on the topic of the article and subject of interest, mentioned keywords were defined. Searches were limited to domestic and foreign journals and reference books. The inclusion criteria were the year of publication and the relevance of the title and purpose of the articles to the research topic. The exclusion criteria were studies with additional information, irrelevant topic, studies before 2010, and case reports. Accordingly, 153 English-written articles were selected and studied. All authors collaborated in evaluating the selected articles. An AI specialist, who is the fourth author of the article, initially appraised the articles to choose articles containing valuable, correct, and complete information regarding AI technology. After this phase, 16 articles were excluded from the study. Subsequently, the other three authors evaluated the articles on basis of the accuracy and eloquence of information, and methodology of the study presented in each article. Concerning this phase, 21 articles were excluded. In Figure 2, the methodology of the study is summarized.

## Results

According to the mentioned process of methodology, 116 articles were selected for final analysis and article writing. Among 116 articles evaluated, 2 articles were focused on the overall concept of AI and its subtitles, and 114 other articles were focused on the application of AI in different branches of dentistry. Out of the 114 articles, 16 articles were related to dental pathology, 2 articles were regarding dental diagnosis systems, 16 articles were about dental radiology, 7 articles were related to restorative dentistry, 14 articles were regarding periodontology, 15 articles were about endodontics, 19 articles were related to orthodontics, 7 articles were about prosthodontics, 2 articles were regarding pediatric dentistry, 2 articles were related to dental surgery and 14 articles had a multidisciplinary approach covering various branches of dentistry.

## Discussion

Nowadays, AI's influence in all branches of dentistry is evident. In the following, we discuss the impact of AI on each branch separately. 

### Pretreatment phase

AI technology can perform simple tasks, including booking and coordinating regular appointments according to the convenience of the patients and dentists, and managing the paperwork and insurance. Furthermore, AI can assist in more complicated tasks such as alerting the dentists about any allergies or medical conditions that the patient may have, and also setting up regular reminders for patients who are on tobacco or smoking cessation programs. It is also observed that AI is beneficial in providing emergency teleassistance in cases of dental emergencies when the dental health care professional cannot be contacted, with higher precision and fewer errors [ 10 ]. On the other hand, due to the Covid-19 pandemic, AI technology can screen patients and secure dental staff's health [ 11 ]. In a study by McCall *et al*. [ 11 ], it was elucidated that AI systems can collate data regarding several key risk factors of Covid-19 in order to aid in screening people and monitoring social media, newsfeeds, or airline ticketing systems across the world. The risk factors included in this study were the transmissibility of the virus and risk populations, the natural history of infection, the incubation period, the mortality rate, and the characteristics of the organism in charge. However, the type of AI required to perform this task is not specified [ 11 ].

### Diagnosis and treatment plan

Probably the most prominent effect of AI in dentistry is in the process of diagnosis and decision-making. Many studies have confirmed AI systems can easily integrate data collected from the patient and present a good outcome that helps the dentist with her final diagnosis and treatment plan [ 12 - 16 ]. In order to accomplish this mission, AI systems initially need to be trained with a vast amount of data collected from reliable sources. In other words, valid information about the possible mutual relationship between medications, systemic conditions, and clinical dental manifestations is gathered from textbooks and previous clinical experiences to train AI systems. Moreover, AI learns to consider the effects of a patient's past dental and medical history, patient preferences, and imagery data all together on the final dental outcome and presents the intended treatment plan. In this case, it can be almost assured that all the factors affecting the patient's dental condition have been concluded to deliver the treatment plan [ 12 - 16 ]. Nevertheless, it should be noted that AI application for automated interpretation in practice is extremely costly and requires the dentist's constant careful inspection [ 17 ].

### Radiology

Different forms of AI are used in maxillofacial radiography. For instance, ANN can be applied for classification, detection, and segmentation in the field of radiology. The classification includes a broad spectrum from the detection of the absence or presence of pathology to classifying the type of detected malignancy. Detection is identified as a process of determining intended disease expansion in the tissue or vital anatomical structures. Segmentation is a process that segments different anatomical structures or pathologies in images obtained with various modalities, including plain radiography, CT, MR, and ultrasound images [ 18 - 20 ]. 

To use AI, especially DL, in radiology, it is necessary to provide a considerable high-quality amount of data to train the system. Therefore, the radiologist must go through a data curation process. It means that the raw data collected from radiographs should be first anonymized. In the second phase, the representativeness of the data is checked which defines as choosing a set of data that adequately replicates the larger group of patients. In the third phase, standardization of the data format is conducted in order to ease the information process for the AI system. Minimization of noise and other radiographic errors in the data occurs in the fourth phase. In the fifth phase, the segmentation of the region of interest in order to be scanned by the system is run. Ultimately, in the last phase, the radiologist enters descriptive information regarding the meaning of an image into the system to train the AI. As it is observed, this process is time consumable [ 21 - 22 ]. 

After entering the required data into the system, data augmentation is completed. In this step, the input data is modified to alter and change the data representation while keeping the label the same. In most cases, augmentation is achieved by blurring or skewing an image, modifying the contrast or resolution, flipping or rotating the image, adjusting zoom, and changing the location of a lesion. By this means, the efficacy and accuracy of the results presented by AI are enhanced [ 21 ].

Following data augmentation, the input data divides into three data sets, including training, validation, and test data sets. The training data set is used to train and adjust the parameters of the learning model. The validation data are used to monitor the model's performance during training and search for the proper model. The test data are used to evaluate the final performance of the advanced model. In the end, a developed valid model is defined [ 21 - 22 ].

AI can diagnose a broad range of diseases, including dental caries, periodontal disease, osteosclerosis, odontogenic cysts and tumors, and diseases of the maxillary sinus or temporomandibular joints on a radiology image. In other words, AI technology can suggest a list of differential diagnoses by providing the patient's clinical and radiological records [ 17 ].

Recent studies have investigated the use of DL to diagnose dental caries, periodontal disease, vertical root fracture, periapical pathosis, and cysts and tumors of jawbone on radiology images [ 23 - 26 ]. Moreover, a study performed by Chang HJ *et al*. [ 27 ] showed that the novel hybrid intelligent system combined with DL architecture and the conventional computer-aided diagnosis (CAD) approach demonstrates high accuracy and excellent reliability in the automatic diagnosis of periodontal bone loss and staging periodontitis. In this research, DL models were applied to specify radiographic CEJ level and bone level. After the automatic detection of periodontal bone loss, the staging of periodontitis was carried out by CAD. In this step, the percentage rate analysis of the radiographic bone loss combined the tooth long-axis with the periodontal bone and CEJ levels. Subsequently, CAD receives this information to stage periodontitis automatically. 

In panoramic and CBCT imaging, the DL technique can detect and classify teeth stages with automated CAD outputs, assist dentists in decision-making, and reduce charting time by automatically digitally filling patients' records [ 28 - 29 ]. On the other hand, DL with the CAD system could provide information to dentists for the early detection of osteoporosis based on their panoramic images. In this process, AI detects the reduction in mandibular cortical width and the degree of erosion of the mandibular lower cortex [ 30 - 31 ]. Another usage of DL in radiology is an automatic estimation of bone age done by setting regions of interest in the hands and wrists on radiographs [ 32 ].

AI can also automatically determine 3D cephalometric landmarks based on an inputted algorithm [ 33 ]. For instance, in a study conducted by Neelapu *et al*. [ 34 ], 20 landmarks were targeted for automatic detection, of which 12 landmarks exist on the mid-sagittal. Due to the complex detection of these landmarks by a radiologist, this technology improved the outcome and resulted in a better and faster diagnosis.

DL and ML techniques can enhance the quality of usual films taken by radiologists. In other words, AI technology attempts to reduce the errors, particularly noises, in the film [ 35 ]. In research by Zhang K *et al*. [ 35 ], it was explained that ANN models could be trained to reduce the noise in the radiographic film. In order to achieve this aim, ANN models were trained with 400 images of size 180×180. The training images included both before and after the Gaussian de-noising process images. Therefore, in this scenario, ANN models learn the method used in the Gaussian de-noising process and can be beneficial in de-noising of input radiographic films.

Moreover, AI can reduce artifacts caused by metal restorations, including crowns and implants on CT and CBCT images. The proposed methodology for this goal is similar to the methodology used in reducing the noise in the radiographic films mentioned above [ 35 ].

Forensic dentistry is a science that often necessitates accurate and detailed information displayed on dental radiographs. According to poor dental image quality and the possible presence of artifacts and other errors, the application of AI can overcome these difficulties and lead to greater clinical decisions [ 37 ].

### Periodontology

AI technology has prominently advanced in the field of periodontology in recent years. A number of studies have reported the benefits of AI systems in assessing periodontal health and diagnosis of disease [ 38 - 40 ]. For instance, Lee *et al*. [ 41 ] claimed that using DL technology can be more effective than ANN to detect periodontally compromised teeth on periapical radiographs. Moreover, it has been shown the accuracy of detection was higher in premolars than in molars, which are most likely related to the simpler anatomy of the roots in premolar teeth.

On the other hand, another study performed by Yauney *et al*. [ 42 ] showed that ML models could be trained to correlate information derived from clinical examination, dental radiographs, and patient's medical history to diagnose the disease automatically in the future.

One of the newest advancements of AI technology in periodontology is the ability of this system to detect the severity of periodontal disease. In a study conducted by Papantanopoulos *et al*. [ 43 ], it was mentioned that ANN could distinguish between aggressive and chronic periodontitis by assessing immunologic parameters in patients. The accuracy of this methodology was 90-98%, which is statistically valuable.

A novel systematic review study revealed that the accuracy of AI applications in detecting dental plaque is 73.6% to 99%. Nonetheless, the accuracy of this technology in the detection of periodontitis based on intraoral radiographs is reported to be between 74% and 78.20%. Therefore, with the development of AI, it is expected to witness the usage of this technology as a powerful diagnostic tool in periodontology [ 44 ]. 

AI technology has made an enormous impact on dental implantology science [ 45 ]. AI technology is applied to optimize the design of dental implants. In this case, AI models modify the dental implant's porosity, length, and diameter, leading to minimized stress at the implant-bone interface and improved dental implant design [ 46 ]. On the other hand, multiple studies have reported that models can predict dental implant success and osteointegration [ 47 - 49 ]. However, the input data differed among various studies [ 47 - 49 ].

Bone level and the amount of bone that supports dental implants are critical factors in treatment success. It is suggested AI technology, specifically DL, can identify the bone level on radiograph images. This finding can significantly reduce failure in the field of implantology [ 50 ].

Probably, the latest advancement of AI is its ability to identify dental implant brands and the stage of treatment explicitly to ensure efficient care. In one research by Sukegawa *et al*. [ 51 ], the accuracy of DL models in identifying dental implant brands and the stage of treatment based on panoramic radiographic films was surprisingly high. Furthermore, it was noted that multi-task learning facilitated analysis accuracy.

### Oral Pathology

Oral pathologies, including oral lesions and malignancies, are among the most concerning dental problems that need early detection. Nowadays, AI technology has eminently progressed to aid in diagnosing oral pathologies [ 52 ]. 

In a study performed by Mahmood *et al*. [ 52 ], 11 studies that applied AI technology to point out malignant cells based on their distinct histopathologic characteristics were supervised. These studies reported the high accuracy rate of AI technology in detecting different oral lesions, as shown in Table 1 [ 53 - 63 ]. Hence, due to the high risk of bias and probability of accuracy overestimation, AI technology cannot fully be trusted to detect oral pathologies and needs further advancements.

**Table 1:** Accuracy of AI technology in the detection of oral pathologies

Author(s)	AI Technology	Oral Lesion	Study Method	Accuracy of Methodology
Baik *et al*. [ 53 ]	Trained algorithm	Oral Epithelial Dysplasia	Training a sequence of classifiers using morphometric data calculated on nuclei from 29 normal, 5 carcinomas in situ and 28 SCC specimens	80% at the cellular level and 75% at the tissue level
Krishnan *et al*. [ 54 ]	Hybrid Intelligent System	Oral Submucosa Fibrosis	Grading the histopathological tissue sections into normal, Oral submucosal fibrosis without Dysplasia and Oral submucosal fibrosis with Dysplasia	95.7%
Krishnan *et al*. [ 55 ]	ANN	Oral Submucosa Fibrosis	Segmentation of collagen fibers in the subepithelial connective tissue	91.64%
Krishnan *et al*. [ 56 ]	ML	Oral Submucosa Fibrosis	Segmentation and classification of subepithelial connective tissue cells except endothelial cells in oral mucosa of normal and oral submucosa fibrosis conditions	88.89%
Krishnan *et al*. [ 57 ]	Trained algorithm	Oral Submucosa Fibrosis	Delineation of the epithelial layer from histological images in discriminating normal and oral submucous fibrosis	98%
Mookiah *et al*. [ 58 ]	ANN	Oral Submucosa Fibrosis	Detection of textural features of the oral mucosal epithelium to discriminate between normal and oral submucous fibrosis	96.43%
Das *et al*. [ 59 ]	ANN	Oral Squamous Cell Carcinoma	Identification of architectural variations of epithelial layers and the presence of keratin pearls in microscopic view	96.88%
Rahman *et al*. [ 60 ]	ML	Oral Squamous Cell Carcinoma	Analyzing abnormality based on textural features present in squamous cell carcinoma histological slides	100%
Sun *et al*. [ 61 ]	ML	Oral Squamous Cell Carcinoma	Automatic color-based feature extraction system for parameter estimation of oral cancer from optical microscopic images	Not mentioned
Lorsakul *et al*. [ 62 ]	Trained algorithm	Oral Squamous Cell Carcinoma	Simultaneous analysis of multiple biomarker expressions within a single tissue section stained with an immunohistochemistry duplex assay	91.64%
Fouad *et al*. [ 63 ]	ML	Oropharyngeal Squamous Cell Carcinoma	Segmentation of oropharyngeal cancer tissue into epithelial and stromal regions	81%

In an exciting review study, different AI techniques for identifying cancerous oral lesions were examined. In the first method, AI algorithms were trained on hematoxylin and eosin (H&E)-stained tissue sections to detect oral squamous cell carcinoma's specific characteristics, including keratin pearls. In the second method, AI models were trained to overlap cancerous areas in pathologic sections taken from different patients to define the similarities. These similarities were specified as cancer's characteristics for the AI's interpretation accordingly. The results showed a negligible variance between the two techniques, and an acceptable statistical result using either method was observed [ 64 ]. AI technology may also be used to follow up the healing process of the oral lesion based on their radiographic view [ 65 ]. For instance, Yang *et al*. [ 65 ] exhibited that ANN can be used to compare pairs of periapical radiographs before and after treatment and classifies the healing process of lesions. In this study, 196 periapical images before and after dental treatment with one of the labels of "getting better", "getting worse" or "have no explicit change" were extracted to train ANN models.

### Prosthodontics

AI technology in the field of fixed prostheses has advanced extensively. In the modern world, AI models are used to enhance crown designs with better marginal adaptation, prediction of crown longevity, and color reproduction [ 66 ]. 

One of the most important reasons for the crown's failures is a lack of marginal adaption. With the evolution of AI technology, the accuracy of marginal adaptation has significantly increased [ 67 ]. For instance, a study revealed that using intrinsic AI and algorithms for automatic tracing of the margin line in the implant-abutment through subgingival could improve marginal adaptation by 96.2%. Nevertheless, this finding was exclusively related to monolithic zirconia crowns and might not be valid for other types of crowns. Due to the absence of studies regarding AI's efficacy in marginal adaptation improvement in crowns excepting monolithic zirconia crowns, the mentioned methodology can be tested in other types of crowns in future research [ 68 ].

AI technology can also be used as an assessment tool. Yamaguchi *et al*. [ 69 ] evaluated the probability of CAD/CAM composite resin crowns' debonding using DL. In this study, 2-dimensional images captured from 3-dimensional stereolithography models of a die scanned by a 3-dimensional oral scanner were used. A total of 8640 images from 24 cases including 12 trouble-free and 12 with the debonding problem were collated to train the system. The results showed that AI technology presents a good performance in assessing the likelihood 

of composite resin crowns' debonding.

The human eye has many visual errors and gets tired quickly, which can cause the shade selection process of a crown difficult. Researchers have attempted to implement a system that can keep its accuracy in the long term for many years. Fortunately, in a study, it has been witnessed ANN produces greater accuracy in color reproduction within the given color space than the traditional visual approach. In order to train ANN models, 43 metal ceramic samples were produced by mixing proportional porcelain powders. Following AI's training phase, the color reproduction of 10 different maxillary incisors was done in both AI and visual approaches. In the end, color distributions of target teeth and fabricated metal ceramic specimens by AI were compared [ 70 ].

Identifying dental arch based on Kennedy's classification is crucial for correct treatment planning in edentulous patients. It is seen utilizing ANN can lead to prompt and precise detection. Moreover, one study reported that this accurate classification of a patient's dental arch is higher in the maxilla than the mandible [ 71 ].

It should be noted the side effects of prosthetic treatments would appear in long term. Therefore, the high success rate of AI technology mentioned in current studies cannot be reliable, and further research in the future is indeed needed [ 72 ].

### Restorative Dentistry

In all likelihood, the most impressive role of AI in restorative dentistry is the detection of caries. Most studies have shown a significant impact of AI in caries detection and screening by several methods [ 73 - 75 ]. For instance, Geetha *et al*. [ 76 ] revealed ANN could distinguish normal tooth anatomy from dental caries based on oral images taken from the patient. This technology may also detect interproximal caries that are challenging for dentists to detect. The reported accuracy using AI technology was 97.1%. However, no studies have shown that AI technology can predict the severity of caries, and further studies are required. 

AI can also ascertain dental restorations on dental radiographs. In a study by Abdalla-Aslan R *et al*. [ 77 ], it was observed that AI models could specify dental restoration and separate different restorations by shape and distribution of grey values on dental radiographs.

Since streptococcus mutans (*S.mutans*) constitute the majority of dental caries' construction, selecting an appropriate caries excavation method can lead to better caries removement and subsequently lesser *S.mutans* in the mouth post-treatment. In an interesting study conducted by Javed *et al*. [ 78 ], trained ANN was employed to predict post-*S.mutans* based on pre-*S.mutans* and the chosen caries excavation method. Three disparate caries excavation methods including excavation with carbide bur, polymer bur, and spoon excavator were tested. The results showed a high preciosity of 99%. This outcome can assist the dentist in the proper selection of caries excavation method. 

Other advantages of AI technology are determining post-operative sensitivity and predicting dental restoration failure. A controversial study was conducted to train AI in order to predict the probability of post-operative sensitivity based on key factors including the type of restorative material, the location of the carious defect, and the depth of the carious defect. Training the AI was led by information provided in questionnaires completed by dentists regarding the considered origin of 213 cases of post-operative sensitivity. Since the training of AI models was based on the clinical experience of dentists, the result is not completely reliable [ 79 ]. In another study performed by Aliaga *et al*. [ 80 ], it was seen that a case-based learning model could be used to select the appropriate restorative material and hence, predict the longevity of the restoration. This technique appeared to be a valid method in practice. 

### Dental Surgery

ANN has played a significant role in dental surgeries. It is highlighted that orthognathic surgeries can be massively transforming by using AI technology [ 73 ]. For example, in a study by Lu *et al*. [ 81 ], it was witnessed that ANN models improved the accuracy of orthognathic surgery's outcome considered by oral surgeons. In order to accomplish this aim, 30 pre-treatment facial images of orthognathic surgery cases were evaluated by surgeons and predicted post-surgery facial images were produced. In the next step, trained ANN models were applied to modify the predicted post-surgery results. Comparing the actual post-surgery facial images with pre- and post-AI modification of predicted facial images proved that AI intervention enhances the accuracy by more than 80%. Therefore, AI's application can prominently affect decision-making and treatment planning in orthognathic surgeries [ 81 ].

Nonetheless, the efficacy of orthognathic surgery can also be checked by AI using pre- and post-treatment images of patients [ 82 - 83 ]. For instance, a study by Patcas *et al*. [ 82 ] showed that trained AI models could be applied to score facial attractiveness and age appearance based on pre-and post-orthognathic treatment photographs of the patient. However, the accuracy of this methodology was not mentioned. On the other hand, another work conducted by the same author revealed that AI technology with the same training methodology could be utilized to score facial attractiveness in cleft patients based on pre-and post-treatment frontal and profile images. Ultimately, AI evaluation results were compared to human ratings and no statistical difference was observed [ 83 ].

One of the most prevalent side effects of third molar extraction is paresthesia due to surrounding nerve damage. However, this error can easily be avoided by carefully detecting the nerve's position based on the patient's panoramic images. Moreover, DL systems are now frequently employed to predict the possibility of the inferior alveolar nerve damage during the mandibular third molar extraction using panoramic radiographic images before the surgery. In other words, DL models precisely detect the inferior alveolar nerve position on the panoramic images. Based on the proximity of inferior alveolar nerve to mandibular third molar, DL systems predict the possibility of nerve damage during the surgery. The accuracy of AI technology in assessing the possibility of nerve damage after dental extractions has been reported to be 82% [ 84 ].

Several oral lesions require complicated surgeries. These extensive surgeries may involve a majority of the mouth and subsequently damage vital structures and cause irreversible harm. Therefore, AI technology has aided in precisely detecting oral lesions' location on panoramic radiographs before the surgery and increasing the treatment's success rate. In one study, the accuracy of AI was 90.36% in the detection of ameloblastoma and odontogenic keratocytes on dental images [ 85 ].

### Orthodontics

In the modern world, AI technology has rooted in the field of orthodontics. In other words, various orthodontic procedures have been changed due to AI advancements [ 86 - 88 ]. Cephalometric tracing and interpretation are among the most critical stages in orthodontic treatment planning which also has been transformed subsequent to AI's emersion [ 73 ]. However, various studies demonstrate contradictory results regarding AI's superiority rather than the gold standard technique [ 86 - 88 ]. For instance, research conducted by Kim *et al*. [ 88 ] manifested a high accuracy rate of AI's application in cephalometric interpretation compared to manual marking and diagnosis. On the other hand, a study conducted by Kunz *et al*. [ 86 ] exhibited that no statistically significant differences exist between humans' gold standard and the AI's predictions in cephalometric analysis.

Since the detection of anatomic landmarks is a key to successful orthodontic treatment, multiple studies have mentioned AI's ability to detect these landmarks on different modalities of radiographs. The accuracy of AI in different radiographic modalities is demonstrated in Table 2 [ 88 - 91 ].

**Table 2:** Accuracy rate of correct anatomic landmark identification on different radiographic modalities

Author(s)	AI Technology	Cephalometric Image	Accuracy Rate
Dobratulin *et al*. [ 89 ]	DL	Lateral Cephalometric Radiograph	92%
Kim *et al*. [ 90 ]	ANN	CBCT Images	80.4%
Muraev *et al*. [ 91 ]	ANN	Frontal Cephalometric Radiograph	82%

Another essential element in designing a treatment plan for an orthodontic patient is analyzing growth patterns and estimating biological age. Determination of a patient's developmental stage can quickly be done using various methods. One of the most applicable methods is appraising cervical vertebrae stages based on a lateral cephalometric radiograph [ 92 ]. In a study performed by Kök *et al*. [ 93 ], it was cited that ANN could detect different stages of cervical vertebrae growth from stage 1 to 4 and stage 6. However, it is expressed that the accuracy of this technique in the detection of stage 5 remarkably decreases, and it is preferred to use other methods instead. 

Another methodology to estimate the patient's age is identifying the dental stage of the patient based on panoramic radiographs. This method determines the possible age range of the patient. In a study, the accuracy rate of ANN, based on the later methodology, was reported to be 94.15% [ 94 ].

AI technology has also developed to predict orthodontic treatment needs in the future. For instance, Thanathornwong *et al*. [ 95 ] showed AI models could be used to assess the possible future needs for orthodontic treatment in patients with permanent teeth. For this aim, AI models were trained by data acquired from patients between the ages of 14 to 19 with orthodontic treatment needs. Various variables including overjet, overbite, crossbite, and so on were examined and inputted into the system. Therefore, the system was trained to correlate the mentioned variables to future orthodontic needs and present a reliable outcome. In this study, the accuracy rate was 93%-95%. Moreover, Shin *et al*. [ 96 ] exhibited that the DL method can also evaluate future orthodontic treatment needs based on dentofacial dysmorphisms and a malocclusion observed on patients' cephalograms. This study reported a high accuracy rate of 95% as well.

AI technology can also be applied in severe complicated cases of orthodontic treatments [ 97 ]. Wang *et al*. [ 98 ] showed that DL models could detect asymmetry of the maxilla in patients with unilateral cleft lip and palate on CBCT images. Moreover, Chen *et al*. [ 99 ] introduced a novel ML methodology to identify maxillary structure variation in unilateral canine impaction based on CBCT images. 

### Endodontics

Since endodontic treatment aims to retain the teeth in their functional position and prevent any subsequent complications, AI advancement in this field can be exceptionally effective [ 100 ]. The frequent benefits of AI application in endodontics are mentioned as analyzing root canal anatomy, detecting root fractures, and periapical lesions, precise assessment of working length, predicting the viability of dental pulp stem cells, and predicting the success rate of retreatment procedures [ 101 ].

An accurate working length determination is one of the most important keys to a successful endodontic treatment. Radiographic images are the most prevalent method employed to measure working length precisely [ 102 ]. Recently, some studies have reported that ANN models have aided endodontists in correct working length measurement by automatic apical foramen identification on a periapical image. The accuracy of ANN models was between 93 and 95% [ 103 - 104 ]. 

An essential advantage of AI is detecting periapical lesions and vertical root fractures prior to treatment [ 102 ]. Although AI technology can identify periapical abnormalities in different radiographic modalities, authors have a controversy about this technique's accuracy [ 105 - 107 ]. Therefore, the possible application of AI for periapical lesions detection in the future should be further examined [ 102 ]. On the other hand, a few studies declared a high accuracy rate of AI in detecting vertical root fractures based on patients' radiographic images [ 108 - 109 ]. In one study, this accuracy rate was reported to be 70% in periapical radiographs and 96.6% in CBCT images [ 108 ]. This finding can prevent failures before treatment initiation [ 102 ]. 

Teeth manifest various root anatomies in different people and races. Hence, complete knowledge of these variations is crucial for dentists in treatment. In recent years, frequent subcategories of AI have aided dentists in the flawless diagnosis of root canal anatomy [ 102 ]. For instance, Hiraiwa *et al*. [ 107 ] studied the application of DL to detect the presence or absence of an extra root in the distal root of the mandibular first molar on panoramic images. The gold standard in this study was the dentist's observation and assessment based on CBCT images. The results showed an accuracy of 86.9% compared to the gold standard method. Therefore, with the application of AI, errors caused by a misdiagnosis of root canal anatomy are decreased [ 102 ]. 

A novel research topic that has attracted much attention worldwide is predicting the viability of dental pulp stem cells. Due to stem cells' ability to differentiate into various cells, maintaining these cells' vitality is crucial [ 110 ]. In a study conducted by Bindal *et al*. [ 110 ], it was reported hybrid intelligent system could efficiently estimate the impact of platelet concentrates on the proliferation of stem cells derived from the human tooth. In order to train the system, the data collected from dental pulp stem cells' viability rate subsequent to stem cells' culturing in three different cultures enriched with platelets were used. 

After endodontic treatment, frequent variables can impress the outcome. Lately, ML has been used to assess multiple variables influencing endodontic surgery outcomes and ultimately predict treatment's success rate. The variables employed by different studies to train ML models include tooth type, lesion size, type of bone defect, root filling density, root-filling length, and the apical extension of post, the age, and the sex. The results of these studies suggest the application of ML in assessing the prognosis of endodontic treatment [ 111 - 112 ]. 

Surprisingly, AI has been used in genetics when it comes to endodontics [ 113 ]. In a study by Poswar *et al*. [ 113 ], it was observed that ANN could be used to analyze the gene expression of the two most common periapical lesions, radicular cysts, and periapical granulomas. This study showed that not only the inflammatory nature but also other biological processes might differentiate radicular cysts and periapical granulomas.

### Pediatric Dentistry

Pediatric patients require unique conservations and treatments due to the presence of deciduous teeth. Deciduous teeth have smaller crowns with huge pulps and can be missed or over-retained and interfere with the expected evolution and eruption of permanent teeth. Therefore, early diagnosis of deciduous teeth problems is critical [ 114 ].

A study conducted by Zakirulla *et al*. [ 114 ] concluded that ANN models could be used to detect dental caries and impacted teeth on different modalities of radiographic images in a pediatric patient. Moreover, Kılıc *et al*. [ 115 ] used the DL approach for the automatic detection and numbering of deciduous teeth in children based on panoramic radiographs. The accuracy rate reported in this study was 95%.

### Disadvantages of AI

Despite the mentioned advantages of AI, it should be noted that AI still has three major disadvantages to encounter. First, this novel technology should be simplified to use. In this case, the need for dentists to pass training courses to learn to work with AI systems is eliminated. Second, the required equipment is not economically viable. Third, since the function of AI is yet not completely identified, the presented outcomes cannot be fully trusted, and an experienced dentist must supervise its results. Therefore, prior to the immense application of AI in dental practice, mentioned cons of AI need to be efficiently managed [ 116 ].

## Conclusion

With the significant development of AI, all branches of dentistry have also benefited from its desirable changes. Today, integrating these two sciences has led to improved detection of dental abnormalities and oral malignancies based on radiographic view and histopathological features, enhanced designing of dental implants and crowns, precise tooth preparation finishing line determination, accurate analysis of patient's growth patterns, correct biological age estimation, better prediction of the viability of dental pulp stem cells, gene expression of periapical lesions analysis, and reliable prediction of the success rate of different dental treatments. These mentioned outcomes can ultimately result in enhanced diagnosis and treatment planning, reduced time waste, and greater patient satisfaction. However, it should be noted that this new technology has important challenges to face. Therefore, the application of AI in clinical dentistry can be considered a major contributing factor to higher success rates and fewer treatment failures in the future only after solving its basic problems.
